# Gender disparity in critical care publications: a novel Female First Author Index

**DOI:** 10.1186/s13613-021-00889-3

**Published:** 2021-07-02

**Authors:** Sowmya Chary, Karin Amrein, Djøra I. Soeteman, Sangeeta Mehta, Kenneth B. Christopher

**Affiliations:** 1 Biogen, Inc., 225 Binney St, Cambridge, MA 02142 USA; 2 Division of Endocrinology and Diabetology, Medical University of Graz, Auenbruggerplatz 15, 8036 Graz, Austria; 3 Center for Health Decision Science, Harvard T.H. Chan School of Public Health, 677 Huntington Avenue, Boston, MA 02115 USA; 4 Sinai Health System, and Interdepartmental Division of Critical Care Medicine, University of Toronto, 600 University Ave, Toronto, ON M5G 1X5 Canada; 5 Division of Renal Medicine, Channing Division of Network Medicine, Brigham and Women’s Hospital, Harvard Medical School, MRB 418, 75 Francis Street, Boston, MA 02115 USA

**Keywords:** Gender, Authorship, Critical care, Gender bias, Gender parity

## Abstract

**Background:**

Bibliometric analyses show gender bias against women in scientific publications and citations. We hypothesized that a metric of an individual senior author’s inclusivity of women as first authors in critical care publications would predict gender inequality.

**Methods:**

Using PubMed and Web of Science, we conducted a bibliometric analysis of original research publications in critical care from 2008 to 2018 in 11 specialty and general journals. Gender for first and senior authors was assigned by a gender determination application, and manually if needed. For all senior authors we defined the novel Female First Author Index (FFA-index) = #Female first authors in publications by an individual senior author/Total # publications by that senior author. We produced a novel interactive web-based application using the R package Shiny to increase potential utilization of the FFA-index.

**Results:**

Of 7370 publications, 30.4% had female first authors and 15.5% had female senior authors. After adjustment for impact factor, journal, year of publication, number of authors, country, and gender determination accuracy, female senior authorship was associated with a 1.9-fold increase in female first authorship [OR = 1.85 (95% CI 1.62, 2.11); *p* < 0.001] compared with male senior authorship. The mean (SD) FFA-index for all individual senior authors was 30.5 (42.9); with a significant difference in FFA-index between male and female senior authors (27.6 versus 42.5, respectively; *p* < 0.001). The interactive web-based application (FFA-index App) produces the same FFA-index output as our study results.

**Conclusions:**

Female representation at prominent authorship positions in critical care publications is still far from achieving gender parity. By creating an authorship index score, we propose a frame of reference for the advancement of female first authorship.

**Supplementary Information:**

The online version contains supplementary material available at 10.1186/s13613-021-00889-3.

## Background

Women are under-represented in critical care training programs, attending/consultant positions, academic faculty, as well as positions of leadership [1]. A majority of women critical care medicine faculty are shown to be personally or professionally impacted by gender inequality [2, 3]. This gender inequality manifests on medical panels, task force participation, guideline publications, conferences and in editorial board positions [4]. Women in general and specifically in the field of critical care medicine commonly have a less robust publication record when compared to their male counterparts [5–8].

Original, peer-reviewed, research publications are crucial for peer recognition and academic professional advancement. As a measure of productivity in research, publication metrics are used for decisions on tenure, promotion, grant funding and university resource allocation. In science, women are less frequently invited by journals to submit commentaries or manuscripts, and less able to secure grants than their male colleagues [9–11]. Of the top 50 cited clinical research articles on sepsis published between 1974 and 2008, only four have a female first author [12].

Our overall aim was to assess a given senior author’s inclusivity of female authors in the prestigious first authorship position. We hypothesized that senior author gender would be an important predictor of first author gender. We also hypothesized that a metric of an individual senior author’s inclusivity of women as first authors in critical care publications would predict gender inequality. To identify the gender difference in authorship as well as the magnitude and trends of disparity we performed a retrospective bibliometric analysis of original research publications from 11 journals over a 10-year period. To measure the contribution of senior authors to female first authorship, we created a metric of the proportion of female first authors in an individual senior author’s publication record.

## Methods

We performed a retrospective bibliometric source analysis of original research publications in the field of critical care medicine on 14,846 publications from 11 journals: New England Journal of Medicine, The Journal of the American Medical Association, The Lancet, British Medical Journal, Critical Care, Intensive Care Medicine, Critical Care Medicine, CHEST, American Journal of Respiratory and Critical Care Medicine, Annals of Intensive Care, and Journal of Critical Care. Journals were selected for their high impact factor and their importance in publishing studies in the field of critical care. As a bibliometric analysis, the authorship data utilized are publicly available and there were no patients involved in this study. IRB review and approval was not required.

We conducted a detailed database search on PubMed of 14,846 publications for citations of all original articles published between January 01, 2008 and December 31, 2018. Publications were selected for analysis based on predetermined inclusion criteria: (a) original research studies including clinical trials, observational studies (including systematic reviews and meta-analyses) and experimental studies that included human samples; (b) studies published between the specified time period; (c) studies published in one of the 11 pre-specified journals, and (d) studies conducted in the field of critical care medicine. All publications in Critical Care, Intensive Care Medicine, Critical Care Medicine, Annals of Intensive Care, and Journal of Critical Care that met these criteria were included. For the New England Journal of Medicine, The Journal of the American Medical Association, The Lancet, British Medical Journal, CHEST, and American Journal of Respiratory and Critical Care Medicine, we also utilized Medical Subject Headings (MeSH) terms related to critical care to identify and include publications (Additional file 1).

**Fig. 1:**
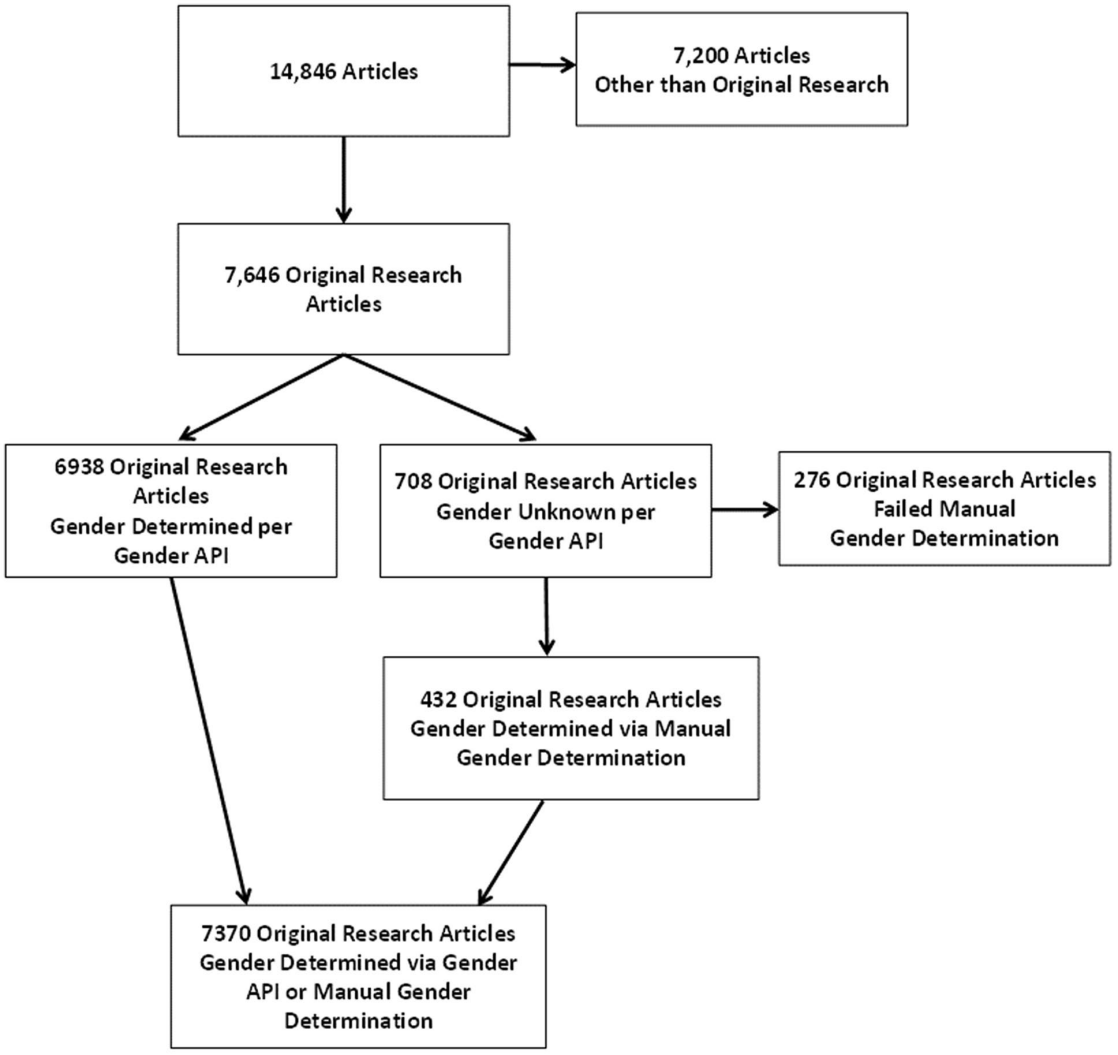
Flowchart of Authorship Gender determination of original articles

Case reports, case-series, letters to the editor, commentaries, guidelines, narrative reviews and single author publications were excluded from our analysis utilizing PubMed tools and a manual approach. Guideline publications were excluded as they are not original research publications, usually including established authors. We excluded 7200 publications which did not meet our selection criteria (Fig. 1), which left 7646 eligible publications. We parsed the bibliographic information output to generate data for the following variables of interest: total number of authors, names of the first and senior author, journal name, year of publication, PubMed ID, type of study, and impact factor of journal at time of publication. First authors were defined as the author whose name was listed first in the publication and senior authors defined as the author listed last. For group publications, the author listed last in the PubMed author list was considered the senior author.

We utilized PubReMiner, a data-mining, front-end, web-based, meta-tool of the PubMed database. With PubReMiner, we categorized the PubMed results according to various strata (full author names, PubMedID, year, journal, type of study, etc.) [13, 14]. To identify country of academic affiliation for the first authors we used the R package easyPubMed. Using the full author names, gender was then categorized for the first and senior author of each article using an application programming interface (API) gender determination platform called Gender API—see Additional file 1 for more details [15].

If gender was not provided for a given author name by Gender API, we utilized a manual determination strategy to determine the gender of the authors using web-based platforms such as Research-Gate, Google Scholar, Google images, LinkedIn, and academic institutional websites. For a given citation, if gender was not provided for a given name by Gender API and gender could not be determined with our manual approach, we omitted the publication from our analysis. To validate the accuracy of the Gender API assignment, two independent researchers, who were blind to the Gender API output, each manually determined the gender of 1000 different first and senior authors, the outcomes of which were then compared to the gender assignment from Gender API.

For all unique senior authors, we calculated the novel Female First Author Index (FFA-index) = #Female first authors in publications by an individual senior author/Total # publications by that senior author. The FFA-index expresses the proportion of female first authors in the critical care related publication record of an individual senior author. A higher FFA-index for an individual senior author reflects a greater proportion of female first authors in the publications by that individual senior author.

Categorical covariates were described by frequency distribution, and compared across authorship groups using contingency tables and Chi-square testing. Continuous covariates were examined graphically and in terms of summary statistics, and compared across authorship groups using one-way analysis of variance (ANOVA) or Kruskal–Wallis equality-of-populations rank test. Unadjusted associations between senior author gender and female first authorship were estimated by bivariable logistic regression analysis. Adjusted odds ratios were estimated by multivariable logistic regression models with inclusion of covariate terms thought to plausibly interact with both senior author gender and female first authorship. All analyses were performed using STATA 14.2MP (College Station, TX).

Finally, to increase the utility of our FFA-index, we designed and implemented an interactive FFA-index tool with the Shiny web-based application framework for R (http://shiny.rstudio.com/). The FFA-index tool combines interactive web interfaces with the Gender API, PubReMiner, and easyPubMed output produced in our bibliometric study. The FFA-index tool determines the FFA-index of a particular senior author based on first name and last (family) name within the 11 journals and 10 years studied in the database. The FFA-index tool is freely available online at the following link: https://kenneth-b-christopher.shinyapps.io/FF_Author_Index/. We tested the FFA-index tool on macOS, iOS and Windows operating systems.

## Results

A total of 7646 publications met inclusion criteria. Of these, 7370 had the gender determined for the first and senior authors and formed the analytic cohort (Fig. 1). Gender API provided gender for first and senior authors in 6938 publications. The Gender API platform was not able to determine gender in 708 publications; of these, 432 had the gender of the first and senior authors successfully determined by a manual strategy. For authors with gender determined by Gender API, the mean (SD) Gender API computed % accuracy for first authors is 96.2 (7.3) and for last authors is 96.4 (7.3). Validation of the Gender API output relative to manual gender determination showed a 100% match.

**Fig. 2:**
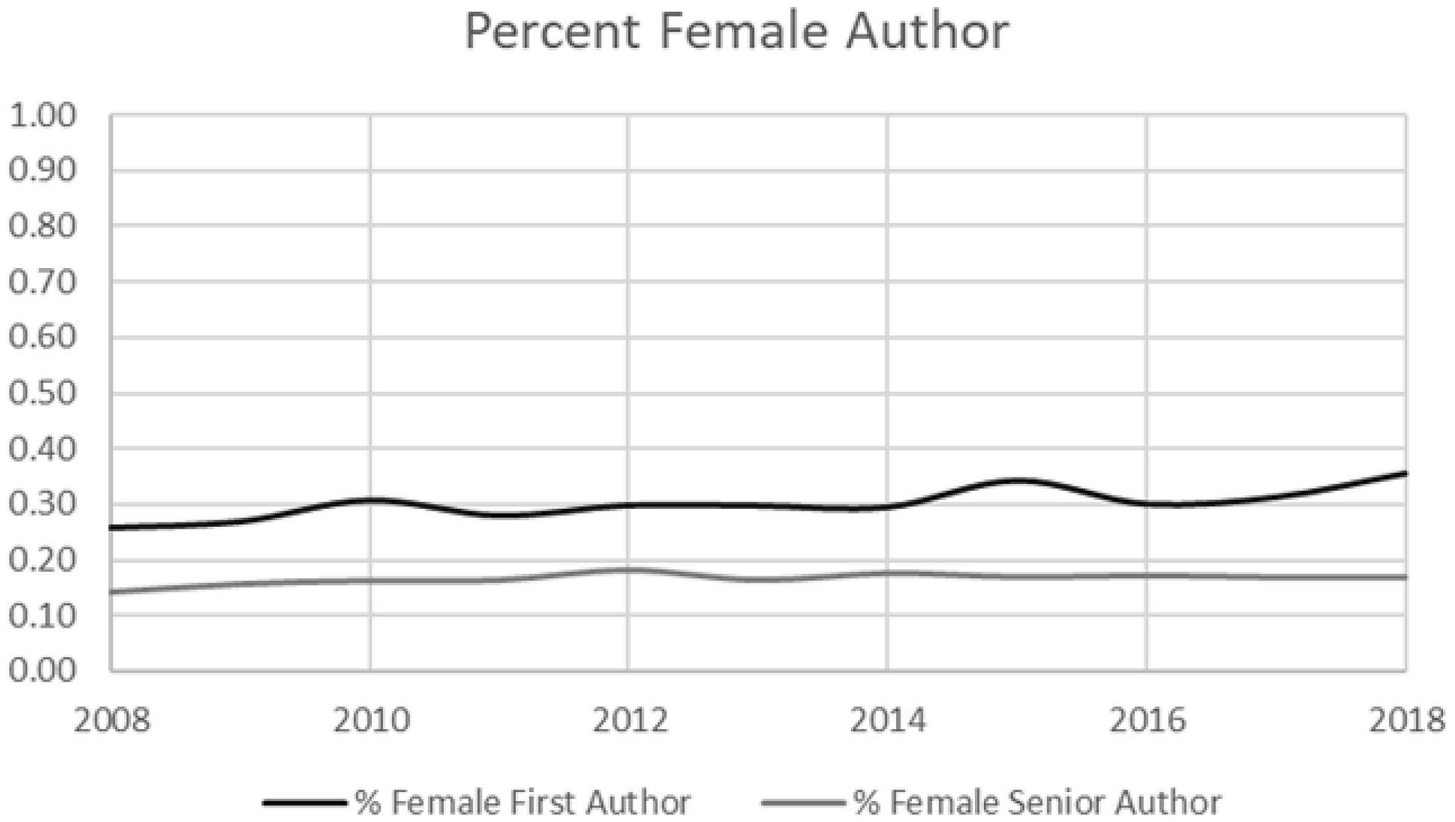
Percent first and senior female authorship over time

There were 5153 different individual first authors identified in the cohort, of whom 34.1% were female. We identified 3890 individual senior authors with 19.6% being female. Of the 7370 publications, 2237 (30.4%) had a female first author and 1140 (15.5%) had a female senior author. The proportion of female first and senior authorship did not increase from 2008 to 2018 (Fig. 2).

The mean (SD) number of authors for manuscripts with female first authors was 7.9 (6.0) and for manuscripts with male first authors was 8.3 (4.2) (ANOVA *p* < 0.001). The impact factor of the 7370 publications ranged from 1.7 to 79.3 with a mean (SD) of 6.2 (6.1). The mean (SD) impact factor for manuscripts with female first authors was 6.0 (5.5) and for manuscripts with male first authors was 6.3 (6.4) (ANOVA *p* = 0.055) which did not change with restriction to higher impact factors (i.e., > 5, > 10, or > 20, data not shown). The median [interquartile range] citations per calendar year for female first authors was significantly lower compared to male first authors at 2.3 [1.0, 4.4] versus 2.6 [1.3, 5.2], respectively (Kruskal–Wallis *p* < 0.001).

Female senior authorship was associated with increased odds of female first authorship [OR = 1.88 (95% CI 1.66, 2.15); *p* < 0.001]. After adjustment for impact factor, journal, year of publication, number of authors, country and Gender API accuracy, female senior authorship was associated with a 1.9-fold higher odds of female first authorship [OR = 1.85 (95% CI 1.62, 2.11); *p* < 0.001] compared with male senior authorship. Though impacted by low relative numbers of manuscripts, only institutions in Finland, Turkey, the Netherlands, and Greece approached gender parity in first authorship; meanwhile, institutions in Germany and Japan had the lowest percent of female first authors, at 20.5 and 6.1, respectively (Table 1). The Pearson’s correlation coefficient between Female First Author percent and FFA-index relative to country is 0.98 with a *p*-value < 0.001.

**Table 1:** Female First Authorship and FFA-index by country

Country	Female First Author (%)	Female First Author Index	Total publications (%)
Finland	56.6	51.6	0.7
Turkey	55.3	53.1	0.6
Greece	48.0	46.4	1.4
Netherlands	45.1	45.7	6.1
Norway	44.2	46.3	0.6
Belgium	43.3	38.4	2.8
Brazil	43.0	39.9	2.3
Sweden	42.4	39.0	1.6
India	35.3	40.5	0.5
UK	32.6	32.6	3.9
Canada	31.8	30.2	5.3
United States	31.5	32.2	25.1
Switzerland	30.9	32.2	2.2
Denmark	30.7	33.3	1.0
Australia	30.5	29.7	3.9
Israel	29.6	28.6	0.6
Chile	28.1	30.3	0.4
Spain	25.6	26.0	4.2
Italy	25.3	25.3	4.9
France	24.6	24.6	14.6
South Korea	22.6	21.9	0.4
China	22.2	22.7	0.6
Austria	21.4	19.6	1.4
Germany	20.5	20.9	7.5
Czech Republic	19.4	17.9	0.4
Japan	6.1	6.3	2.0

The mean FFA-index for all 3890 individual senior authors was 30.5 (42.9). For male senior authors (*N* = 3125) the mean (SD) FFA-index was 27.6 (41.2); and for female senior authors (*N* = 763) the mean (SD) FFA-index was 42.5 (47.6) (*χ*^2^ = 46.6; *p* < 0.001). It should be noted that the FFA-index was not weighted by geographical variation of the proportion of female intensivists or female academic intensivists. The FFA-index followed a similar pattern of country of academic affiliation as female first authorship (Table 1). For male senior authors with five or more senior author manuscripts (*N* = 251), the mean (SD) and median FFA-index were 28.7 (26.8) and 20.0. For female senior authors with five or more senior author manuscripts (*N* = 20), the mean (SD) and median FFA-index were 47.5 (23.9) and 46.4. These gender differences in FFA-index in those with five or more senior author manuscripts were significant (*χ*^2^ = 12.9; *p* < 0.001). Finally, the R Shiny FFA-index tool performance replicated exactly the FFA-index for individual senior authors, male senior authors and female senior authors.

## Discussion

Similar to others, we find that first and senior authorship positions in original research publications in critical care medicine is far from gender parity [16]. With the development of the FFA-index, we demonstrate that in critical care, only female senior authors have achieved gender parity in first authorship. Productive male senior authors show a significant decrease in the FFA-index compared to productive female senior authors. The FFA-index has the potential to serve as a benchmark of senior author inclusivity.

Gender disparity is pervasive in business and academia [17–19]. The World Economic Forum reports that with the current rate of change, gender disparity will persist well into the next century [19]. An important aspect of improving the career progression of women is enhancing their publication record. Bibliometric related indexes including the most commonly used h-index, as well as the m quotient, contemporary h-index and i10 index are strongly positively correlated with academic productivity [20–24]. But such metrics do not account for gender. As PubMed and Web of Science do not index gender, a bibliometric index that accounted for gender existed only in proposal form [25]. The h-index is increased by self-citation, itself known to be more common among men [20, 26]. As in other male-dominated fields, the rate of increase in female senior authorship in critical care is nearly stagnant [9]. Research publications in the field of critical care medicine show overwhelming authorship gender disparity and our analysis over the last decade shows that we are far from bridging this gap [16].

Although previous data show that both male and female faculty members exhibit bias against female undergraduate students [27], similar to Vranas et al., our analysis in the field of critical care showed female senior authorship to be associated with a 1.9-fold higher likelihood of female first authorship following adjustment [16]. Assessing disparity at the authorship level versus the journal level may be important in identifying modifiable contributors to gender disparity. Also, the awareness of one’s own publishing pattern as a senior author may increase inclusive behaviors that promote female first authorship. Thus, we designed the novel critical care-based FFA-index to evaluate the proportion of female first authors in an individual senior author’s original research publication record. A senior author’s publishing pattern is potentially modifiable but may be steeped in assumptions, stereotypes and influenced by the academic climate [28, 29]. By creating the FFA-index, we have established a frame of reference for female first authorship. Senior authors across the globe can now use the FFA-index to assess their own contribution to achieving gender equality in the field of critical care. If the construct validity of the FFA-index is confirmed outside of critical care, the FFA-index has the potential to be utilized as a key performance indicator of gender parity in authorship.

Academic journals have an important role to play in achieving gender parity. Double-blind peer review, though applied infrequently, allows for the research content to achieve prominence rather than judgment of authors’ names, gender, institution, country, language or past publication success [30, 31]. A randomized trial comparing single versus double-blind peer review of orthopedics original research papers demonstrated a significantly higher acceptance rate and perceived quality when prestigious author and institution information is included [32]. The institution of a universal policy of double-blind peer-review, which appears to have broad support among the research community, may reduce bias against women in the peer-review process [33, 34].

Critical care medicine is at high risk of perpetuating gender disparity due to implicit gender bias [35]. Individual institutions should address gender bias. Educational interventions and bias reduction strategies can reduce implicit gender bias against female trainees [36]. Efforts should also focus on development of the requisite research skills and knowledge by supporting robust cost effective clinical and experimental research methods and skill training programs. Higher FFA-index scores may result from incentivization of senior authors to strive for inclusiveness of female first authors. Finally, institutional sponsorship of female first authors can serve to enhance academic opportunities for women [37, 38].

Ours and other data indicate that gender inequality exists in critical care publications [16]. While the data show that female first and senior author publication rates have stagnated for the last 10 years, there are some notable positive developments in gender parity in critical care leadership and in societies’ commitments toward increased representation [38]. Organizational change is catalyzed by practice and policy change. Our gender parity metric (FFA Index) and web-based interactive tool will hopefully promote a more open fact-based conversation on the next steps. Without improvements in first female authorship rates to increase accumulated career capital, it is estimated that true gender parity will not exist in the critical care research community for another 50 years [9]. The FFA-index can be utilized for individual and institutional goal-setting for higher female representation.

The present study is unique in that it provides an individual-level metric of senior authors on female first authorship. Because the FFA-index is derived from critical care medicine publications, it does not require Web of Science subject category based field*-*normalization [39]. Others have created and utilized index scores derived from bibliometric analyses to study gender disparities in science. A previous study on authorship gender disparities in research publications utilized the Prestige Index derived from all authors to evaluate a given journal’s inclusivity of female authors in prestigious first and senior authorship positions [40–42]. This Prestige Index can be applied to individual journals, fields and geographic areas but not to individual senior authors [42].

Our study has limitations. Utility of the FAA-index is currently limited to critical care research. We only studied articles from journals with impact factors which may have decreased the proportion of female first authors. Journals with impact factors less than six or those without impact factors as a group are noted to have a higher proportion of female first authors [16]. PubReMiner output of names with non-standard English characters (i.e., ö, æ, ø, å) do not interface with Gender API, which required a manual strategy to determine gender. Gender API did not report gender if the accuracy is below 50% which required a manual determination of authorship in 9% of our articles. Despite our additional manual strategy to determine gender, 3.6% of publications could not have gender determined in either the first and last authors due to unisex first names or lack of an online presence. The accuracy of Gender API gender determination depends on government datasets and social media data which may be imprecise. Our exclusion of publications not classified as original research relies on PubMed characterization data which may not be exact. Also, we were unable to evaluate the issue of co-first or co-senior authorship. Further, we were not able to include authors included in a group but not individually listed as part of the PubMed citation. Importantly, our FFA-index does not take into account the geographical variability of the proportion of female intensivists or female academic intensivists. Regarding the FFA-index, we know little about the likelihood that a female first author will have academic success given that they have published with a senior author with a high FFA-index [43]. Finally, gender parity will not be achieved with the FFA-index alone as behavior does not change with awareness in the absence of other interventions [36].

## Conclusion

Over the last decade a significant authorship gender disparity in critical care has persisted. While the number of publications only provide a limited indicator of researcher impact, the proportion of female first authors that an individual senior author publishes with is indicative of inclusivity. Creation of the FFA-index is a potentially important step to address authorship gender disparity. Evaluation of the construct validity of the FFA-index and its performance in other fields will be important in determining the utility of a bibliometric-based metric in advancing women’s academic advancement.

## Supplementary Information

**Additional file 1.** Supplemental Methods.

## Data Availability

PubMed data utilized are publicly available.

## Abbreviations

API: Application programming interface
FFA-index: Female First Author Index
