# Supplementary material for: Gender disparity in critical care publications: a novel Female First Author Index
Source: Ann Intensive Care. 2021 Jul 2;11:103. doi: 10.1186/s13613-021-00889-3 (PMC8253865; doi:10.1186/s13613-021-00889-3)
Supplement: Supplementary file 1 — Additional file 1. Supplemental Methods. [file 13613_2021_889_MOESM1_ESM.docx]

**Gender Disparity in Critical Care Publications: a novel Female First Author Index**

Sowmya Chary, Karin Amrein, Djøra I. Soeteman, Sangeeta Mehta, Kenneth B. Christopher

**Additional Methods**

*Medical Subject Headings (MeSH) terms*

Critical Care

Critical Care/Standards

Critical Illness

Critical Illness/Mortality

Critical Illness/Therapy

Intensive Care Units

Critical Care/methods

Critical care/utilization

Intensive Care Units, Neonatal

Intensive Care, Neonatal

Intensive Care, Neonatal/methods

Intensive Care, Neonatal/utilization

Intensive Care Units, Pediatric

Intensive Care Units

Critical Care/economics

Critical Illness/economics

Critical illness/psychology

Intensive Care unit/Statistics & Numerical Data/utilization

Intensive Care unit/Statistics & Numerical Data

*Gender API*

Using the full author names, gender was then categorized for the first and senior author of each article using an application programming interface (API) gender determination platform called Gender API (1). Gender API is a web-based software that determines gender from names using a previously imputed database produced from publicly available governmental sources and social networks database of approximately 6 million names from 189 countries. Names are automatically searched in a specific country, then globally, and then using spelling normalizations. Gender API uses a multi-layer, cloud-based infrastructure with a 99.9% availability.

Apart from determining the gender of a given name, Gender API also provides an accuracy percentage for each gender ranging from 50-100%. The accuracy measure per gender assignment is dependent upon the number of data points the platform contains for each individual name. If the Gender API platform is unable to meet the requirements for >50% accuracy, it provides an unknown gender for that author name. A reliability analysis of scientific author gender using the five most common and popular gender determining methods found Gender API to generate the most accurate results (2).

References Cited

1. Gender API [Available from: <https://gender-api.com/>.

2. Santamaría L, Mihaljević H. Comparison and benchmark of name-to-gender inference services. PeerJComput Sci. 2018;4:e156.
